# Screening Neurocognitive Disorders in Primary Care Services: The Quick Mild Cognitive Impairment Approach

**DOI:** 10.1093/geroni/igaa057.515

**Published:** 2020-12-16

**Authors:** Constanca Paul, Susana Sousa, Pedro Santos, Rónán O’Caoimh, William Molloy

**Affiliations:** 1 University of Porto, Porto, Portugal; 2 Biomedical Sciences, Abel Salazar Biomedical Sciences Institute - University of Oporto, Porto, Portugal; 3 National University of Ireland, Galway City, Galway, Ireland; 4 St. Finbarr’s Hospital, Cork, Ireland

## Abstract

Neurocognitive Disorders (NCD) is an increasingly common condition in the community. The General Practitioner (GP) in Primary Care Services (PCS), have a crucial role in early detection of NCD and is usually the first professional to detect the signs of MCI. The objective of this study was to test the feasibility and utility of the cognitive screening instrument QMCI in Primary Care. A community sample of 436 people 65+ living in the community was randomly selected from a larger group of old people with mental health concerns (N=2734), referred by their GPs. The mean age of the sample was 75.2 years (sd 7.2), with 41.3% men and 58.7% women; 60.4% married followed by 28.7% widows. The education level was low with 21% illiterate and 69,8% people with 4 years education. The QMCI mean was 37.1/100 (sd 16.2). The amount of people screening positive for cognitive impairment QMCI (<62/100) was 94.2%. In the distribution of people with cognitive impairment by Global Deterioration Scale (GDS) three recoded categories, of the 286 people 76,1% where classified as having very mild or mild impairment, 19,4% moderate or moderately serious and 4,5% severe or very severe impairment. These results confirm the perception of GPs about their clients having mental health concerns and the ability of QMCI accurately discriminate MCI. The QMCI is very brief (3-5mins) fitting the short time of GPs to assess cognitive status and timely refer clients to nonpharmacological interventions that could postpone NCD symptoms.

